# VR/AR Head-mounted Display System-based Measurement and Evaluation of Dynamic Visual Acuity

**DOI:** 10.16910/jemr.12.8.1

**Published:** 2019-10-15

**Authors:** Jung-Ho Kim, Ho-Jun Son, Seung-Hyun Lee, Soon-Chul Kwon

**Affiliations:** Industry-Academic Collaboration Foundation, Kwangwoon University, Seoul, Korea; Strategy and Planning Team, Korea VR AR Industry Association, Seoul, Korea; Ingenium College of Liberal Arts, Kwangwoon University, Seoul, Korea; Graduate School of Smart Convergence, Kwangwoon University, Seoul, Korea

**Keywords:** augmented reality, dynamic visual acuity, head-mounted display, King–Devick test, virtual reality

## Abstract

This study evaluated the dynamic visual acuity of candidates by implementing a King–Devick (K-D) test chart in a virtual reality head-mounted display (VR HMD) and an augmented reality head-mounted display (AR HMD). Hard-copy KD (HCKD), VR HMD KD (VHKD), and AR HMD KD (AHKD) tests were conducted in 30 male and female candidates in the age of 10S and 20S and subjective symptom surveys were conducted.

In the subjective symptom surveys, all except one of the VHKD questionnaire items showed subjective symptoms of less than 1 point. In the comparison between HCKD and VHKD, HCKD was measured more rapidly than VHKD in all tests. In the comparison between HCKD and AHKD, HCKD was measured more rapidly than AHKD in Tests 1, 2, and 3. In the comparison between VHKD and AHKD, AHKD was measured more rapidly than VHKD in Tests 1, 2, and 3. In the correlation analyses of test platforms, all platforms were correlated with each other, except for the correlation between HCKD and VHKD in Tests 1 and 2. There was no significant difference in the frequency of errors among Tests 1, 2, and 3 across test platforms.

VHKD and AHKD, which require the body to be moved to read the chart, required longer measurement time than HCKD. In the measurements of each platform, AHKD was measured closer to HCKD than VHKD, which may be because the AHKD environment is closer to the actual environment than the VHKD environment. The effectiveness of VHKD and AHKD proposed in this research was evaluated experimentally. The results suggest that treatment and training could be performed concurrently through the use of clinical test and content development of VHKD and AHKD.

## Introduction

Humans receive external information through sensory organs, most of which enters through the eyes. In humans, vision plays a more important role than other sensory organs, and leads or supports the other senses[1].

Visual acuity is classified into static visual acuity, which is the ability to view a stationary object at a certain distance, and dynamic visual acuity, which is the ability to view a moving object[2]. Dynamic vision plays an important role in sports and driving[3, 4, 5, 6, 7, 8] where rapid physical responses to changes in surrounding conditions are needed. Since dynamic visual acuity can be improved through training, programs related to dynamic visual acuity training have been developed[9, 10, 11].

Factors affecting dynamic visual acuity can be classified into physical factors related to the measurement system and physiological factors related to the subjects. Physical factors include optotype brightness, speed, irradiation time, and size, while physiological factors include resolution, periphery cognition, and ocular motility. 

The visual function of recognizing moving targets has been investigated in numerous studies. Ludvigh defined the concept of dynamic vision and first reported that visual acuity decreases sharply as the target speed increases.

In general, human life involves viewing the environment while moving, except when reading or performing some types of office work. Thus, viewing moving objects is an important aspect of visual ability, but standard visual acuity tests are not able to measure such visual acuity parameters, and dynamic visual acuity research is hampered by the lack of a generalized measurement system.

Vision is a much more complex and dynamic process than visualization of fixed external information, such as that measured in a vision test. Human visual ability includes contrast sensitivity, color vision, stereoscopic vision, legibility, visual field, accommodation, pursuit movement, saccades, as well as the ability to see. 

Since its inception, virtual reality (VR) and augmented reality (AR) has been applied in various fields, such as medical care, defense, and education. In addition, human visual characteristics in the context of wearing a head-mounted display (HMD) have been analyzed[12, 13, 14].

In the VR/AR HMD environment, information is presented on a display in accordance with the user's gaze and body motion. This allows implementation of targets that are used in actual clinical practice and can provide a dynamic visual acuity test, based on a realistic environment similar to that of the subject, outside of the context of a reading environment.

Due to recent advances in VR technology, targets utilizing VR have been developed[15]. The virtual reality environment is similar to the actual test environment in that it induces body movement of the subject, but has a disadvantage in that the view is blocked. This study implemented a dynamic visual acuity chart that approximates a real-life environment by utilizing an AR HMD and evaluated its effectiveness through comparative analysis between the actual original chart and a VR HMD chart.

This paper is set out as follows. First, the King–Devick test is described, followed by an explanation of the HMD, and then a description of the study subjects and research methods is provided. Thereafter, the experimental results are reported, followed by analysis and discussion of these results, and finally, a concluding statement.

## King-Devick Text

King-Devick Test (KDT), one of the methods for measuring Saccadic Eye Movements, measures the speed in which a person can quickly and accurately read a given number of stimuli[16]. The test is standardized to ages 6-14, but can also be used in adults[17]. The K-D test involves 3 targets (Test I, II, and III), each with 8 lines of 5 numbers per line arranged horizontally but randomly within the target. The numerical size of the target is 20/100 (6/30).

**Figure 1.**
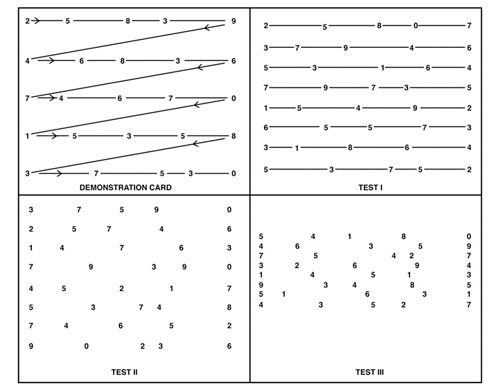
K-D test chart

The tester instructs the testee to read the numbers of each target from left to the right as quickly as possible, and measures the reading time and the number of errors made. A. Cohen and Lieberman (1993) established the standard table for the K-D test through a study of 1,202 students. Table 1 and Figure 1 show the target of the K-D test and the test standard. The K-D test may have limited usefulness in children (6 years or older) with lack of binocular function, and uncorrected refractive error. 

In recent years, the K-D test has been performed not by a hard-copy method, but as a digital test by means of a tablet PC. Standard data are also available for all age groups (www.kingdevicktest.com).

The tablet-based K-D test is equipped with voice recognition and automatic number randomization. Currently, the K-D test is serviced by computers and tablet PC (iOS or Android). The use of hard testing methods has declined over time. 

**Table 1:** K-D test results

**Age ** **(years)**	**Time in seconds** **(total of 3 sub-tests)**	**Number of errors** **(total of 3 sub-tests)**
6	119	17
7	101	12
8	79	3
9	73	3
10	68	2
11	57	1
12	54	1
13	52	1
14	50	0

Source: A. Cohen, S. Lieberman, Report. In: Manual of the NYSOA-KD Saccade Test. Mishawka, Ind: Bernell Corp: 1993.

## Head-Mounted Display

### Optical See-Through Display

In optical see-through displays, the light is transmitted through the HMD display[18]. The light transmitted through the screen is displayed together with the image generated by the computer. Early versions of this type of display had a drawback in that the half-mirror reduced the amount of light transmitted. However, recently developed HMDs solved this problem by using a mini projector and prism. Since the optical see-through method outputs the computer-generated image in a translucent state, it has an advantage that it does not obscure the user's view, but it also has a disadvantage in that it is difficult to match the computer image with the reality observed by the user. In this study, Microsoft’s Hololens was used.

**Figure 2.**
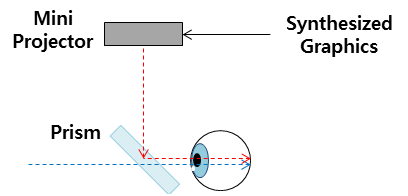
Schematic diagram of an optical see-through display

### Video See-Through Display

Video see-through displays synthesize the images obtained by camera and a computer image and displays them to the user[19]. The video see-through method can be implemented by attaching a camera to the HMD which non-video see-through method.

Since the video see-through method can acquire a greater variety of real-life information than the optical see-through method, and can match this with computer images, it may implement content that allows strong immersion. However, there may be a lag in the camera due to the operation of synthesizing photographic images and graphics.

**Figure 3.**
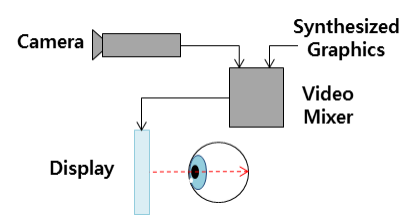
Schematic diagram of a video see-through display

### Non-See-Through display

The non-see-through display blocks the visual field, such that the external environment is not seen, and allows the user to have high immersion. It uses an optical system that can magnify the display at a short distance by means of a lens[20]. This is a popular HMD type, which previously belonged to high-end equipment. The non-see-through HMD tracks the user's position and posture through an infrared sensor or Inertial Measurement Unit sensor. Consequently, the head-tracking speed cannot be accurately tracked, such that a delay or blurring occurs to cause a motion sickness. In this study, the SAMSUNG GearVR and Galaxy Note 3 were used.

**Figure 4.**
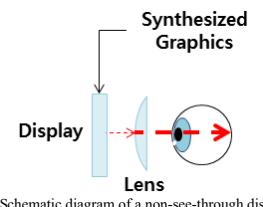
Schematic diagram of a non-see-through display

## Research Subjects and Methods

### Participants

Thirty participants who understood the purpose of this study and had no particular eye diseases or mental illnesses, and had corrected visual acuity of 0.8 or more, were selected as the participants in this research.

### Methods

#### Production of VR/AR HMD-Based Dynamic Vision Test Chart

**Figure 5.**
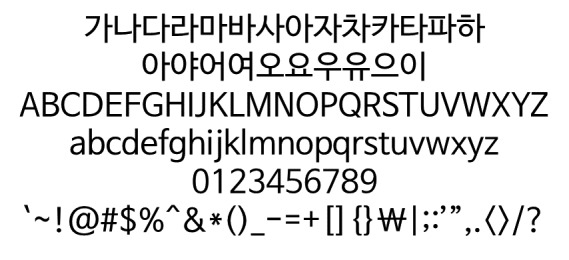
Nanum-Barun gothic font

A K-D test chart, based on a VR/AR HMD used in the test, was produced using Adobe Illustrator and Adobe Photoshop. In the display environment, Namum-Barun Gothic (Bold) was used as a highly readable font (Figure 5) [21, 22].

**Figure 6.**
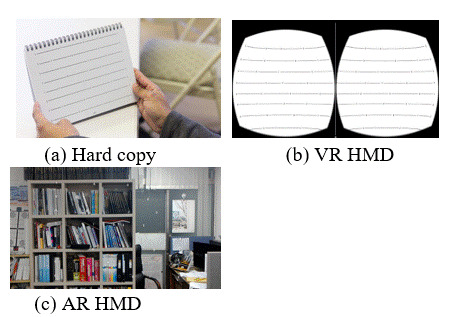
K-D chart for each platform

The character size of the test chart was set based on the Landolt C target, which defines visual acuity and visual angle at 1.0 mm and 1 minute of arc, and can distinguish an internal diameter of 1.5 mm from an external diameter of 7.5 mm at a 5.0-m distance, in accordance with the ratio of the number target[23]. The character size of the VR HMD chart was set at 0.1(6/60, 20/200 feet) the size of a target of 159.31 mm, which is the distance of the VR display (character size: 0.80 × 1.11 mm). Considering the built-in convex lens magnification of 4.43 times for VR HMD, the size of the target output on the display was set as small as the magnification. The character size of the AR HMD chart was set at 0.1(6/60, 20/200 feet) the size of the target in an actual test distance of 3 m (character size: 32.40 × 45 mm). Figure 6 shows an image of the K-D chart for each platform used in the test.

(a) Hard copy (b) VR HMD

(c) AR HMD

In the case of the hard copy K-D (HCKD) chart, the test was performed with the head of the testee remaining stationary, as when reading a book. In the VR HMD K-D (VHKD) chart, all characters were invisible in one field of view and the test required that the subject’s head had to move in order to see the characters. The AR HMD K-D (AHKD) chart was designed such that the characters of all targets were not visible in one view, to induce the testee’s motion. Due to the characteristics of the AR HMD, the test involved observation of a mixture of the K-D chart and real-life space.

#### History Taking and Prior Eye Movement Test

Before starting the test, H-S(Heinsen-Schrock) scale and history taking were carried out to evaluate the physical condition and eye movements of the subject, as these factors affect the test. History taking involved questions about occupation, physical activity, HMD experience, medical history, visual training experience, and driving.

#### Subjective Symptom Survey

After carrying out each test (HCKD, VHKD, AHKD), a subjective symptom survey was administered, which was divided into 6 items, including ① Dizziness, ② Diplopia, ③ Definition, ④ Legibility, ⑤ Discomfort, and ⑥Ocular fatigue. Symptoms were scored subjectively using 4-point Likert scales, with 0 meaning "not at all", 1 meaning "no", 2 meaning "normal", 3 meaning "yes", and 4 meaning "yes indeed." 

Subjective symptoms were measured after randomly performing three tests for each participant.

#### Error measurement

In this paper, we used the error measurement method used in DEM (Development Eye Movement). It is possible to check for errors in the case of substitution error (s), omission error (o), addition error (a), and transposition error (t) while performing KDT.

#### Research Data Analysis

Data were analyzed using paired *t*-tests and simple correlation (Pearson) in SPSS (Ver. 18.0 for Window, SPSS Inc, Chicago, IL, USA). The 95% confidence intervals were calculated, and p < 0.05 was considered to indicate statistical significance.

## Results

### Subjective Symptom Scores

For the question related to dizziness, the mean scores were 0.33 ± 0.66 points, 0.77 ± 1.07 points, and 0.30 ± 0.70 points immediately after the HCKD test, the VHKD test, and the AHKD test, respectively. There was a statistically significant difference between dizziness in the HCKD and VHKD, and between VHKD and AHKD.

For the question related to diplopia, the mean scores were 0.73 ± 1.11 points, 0.80 ± 1.16 points, and 0.80 ± 0.35 points immediately after HCKD, VHKD, and AHKD tests, respectively. There were no statistically significant differences in diplopia.

For the question related to definition, mean scores of 0.77 ± 0.97 points, 1.00 ± 1.14 points, and 0.57 ± 0.94 points were obtained immediately after the HCKD, the VHKD, and AHKD tests, respectively. There was a statistically significant difference in definition between VHKD and AHKD.

For the question related to legibility, mean scores of 0.77 ± 0.86 points, 0.83 ± 1.05 points, and 0.50 ± 0.82 points were obtained immediately after the HCKD, VHKD, and AHKD tests, respectively. The difference in legibility was statistically significant between VHKD and AHKD.

For the question related to discomfort, mean scores of 0.43 ± 0.68 points, 0.40 ± 0.77 points, and 0.30 ± 0.60 points were obtained immediately after the HCKD, VHKD, and AHKD tests, respectively. There were no statistically significant differences in discomfort.

For the question related to ocular fatigue, the mean scores of 0.83 ± 1.15 points, 0.93 ± 1.28 points, and 0.40 ± 0.77 points were obtained immediately after the HCKD, VHKD, and AHKD tests, respectively. There were statistically significant differences in ocular fatigue between HCKD and AHKD, and between VHKD and AHKD.

**Table 2:** Subjective symptoms between K-D tests

						unit: points
**Items**		**HCKD** **M ± SD**	**VHKD** **M ± SD**	**AHKD** **M ± SD**	**t**	**p-value**
① Dizziness	HCKD & VHKD HCKD & AHKD VHKD & AHKD	0.33 ± 0.66 0.33 ± 0.66 -	0.77 ± 1.07 - 0.77 ± 1.07	- 0.30 ± 0.70 0.30 ± 0.70	-2.149 0.254 2.454	0.040 0.801 0.020
② Diplopia	HCKD & VHKD HCKD & AHKD VHKD & AHKD	0.73 ± 1.11 0.73 ± 1.11 -	0.80 ± 1.16 - 0.80 ± 1.16	- 0.80 ± 0.35 0.80 ± 0.35	-0.403 -0.320 0.000	0.690 0.752 1.000
③ Definition	HCKD & VHKD HCKD & AHKD VHKD & AHKD	0.77 ± 0.97 0.77 ± 0.97 -	1.00 ± 1.14 - 1.00 ± 1.14	- 0.57 ± 0.94 0.57 ± 0.94	-1.045 0.947 2.149	0.305 0.351 0.040
④ Legibility	HCKD & VHKD HCKD & AHKD VHKD & AHKD	0.77 ± 0.86 0.77 ± 0.86 -	0.83 ± 1.05 - 0.83 ± 1.05	- 0.50 ± 0.82 0.50 ± 0.82	-0.304 1.439 2.163	0.763 0.161 0.039
⑤ Discomfort	HCKD & VHKD HCKD & AHKD VHKD & AHKD	0.43 ± 0.68 0.43 ± 0.68 -	0.40 ± 0.77 - 0.40 ± 0.77	- 0.30 ± 0.60 0.30 ± 0.60	0.226 1.072 1.795	0.823 0.293 0.083
⑥ Ocular fatigue	HCKD & VHKD HCKD & AHKD VHKD & AHKD	0.83 ± 1.15 0.83 ± 1.15 -	0.93 ± 1.28 - 0.93 ± 1.28	- 0.40 ± 0.77 0.40 ± 0.77	-0.432 2.282 2.641	0.669 0.030 0.013

SD: standard deviation

**Figure 7.**
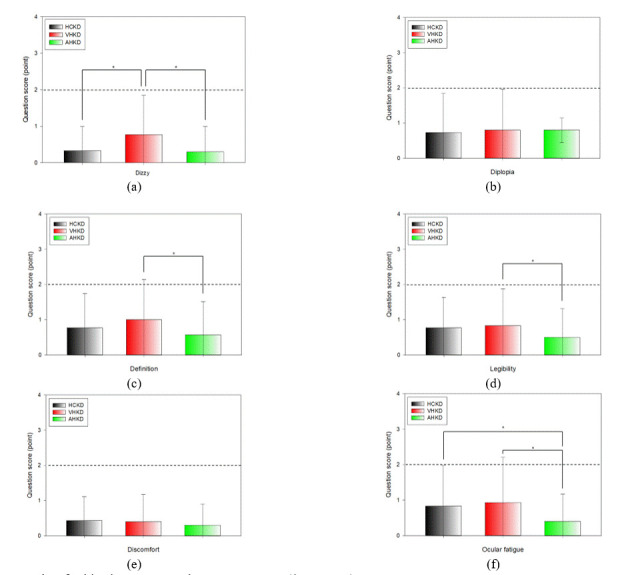
Results of subjective symptoms between K-D tests(*: p < 0.05) (a) Dizziness, (b) Diplopia, (c) Definition, (d) Legibility, (e) Discomfort, (f) Ocular fatigue

### K-D test results

#### Comparison of Results between HCKD and VHKD

Table 3 compares the results of Test 1, Test 2, and Test 3 of the K-D test between HCKD and VHKD. There was a statistically significant difference in the results of Test I between HCKD (12.36 ± 2.06 s) and VHKD (22.24 ± 3.27 s) and HCKD was measured more rapidly. Similarly, for Test 2, there was a statistically significant difference between HCKD (12.90 ± 2.70 s) and VHKD (23.23 ± 3.80 s), and HCKD was measured more rapidly. Furthermore, for Test 3, there was also a statistically significant difference between HCKD (13.89 ± 2.56 s) and VHKD (24.04 ± 3.88 s), and HCKD was measured more rapidly

**Table 3:** Comparison between HCKD and VHKD

				unit: sec
	**HCKD** **M ± SD**	**VHKD** **M ± SD**	**t**	**p-value**
Test 1	12.36 ± 2.06	22.24 ± 3.27	-15.441	p < 0.001
Test 2	12.90 ± 2.70	23.23 ± 3.80	-13.557	p < 0.001
Test 3	13.89 ± 2.56	24.04 ± 3.88	-15.392	p < 0.001

SD: standard deviation

**Figure 8.**
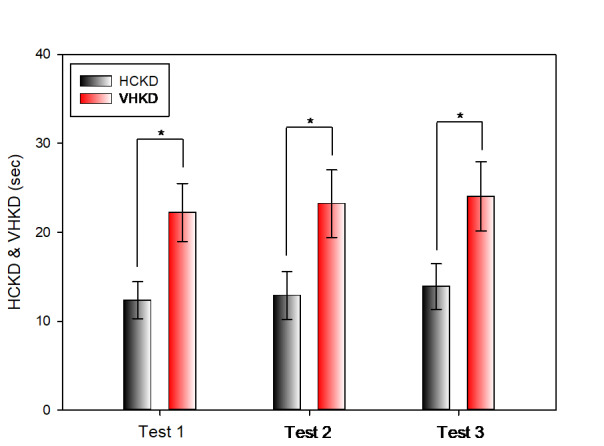
Comparison between HCKD and VHKD(*: p < 0.05)

#### Comparison of Results between HCKD and AHKD

Table 4 compares the results of Test 1, Test 2, and Test 3 of the K-D test between HCKD and AHKD. In Test 1, there was a statistically significant difference between HCKD (12.36 ± 2.06 s) and AHKD (14.50 ± 2.80 s) and HCKD was measured more rapidly. In the case of Test 2, the difference between HCKD (12.90 ± 2.70 s) and AHKD (14.61 ± 2.90 s) was statistically significant and HCKD was measured more rapidly. Similarly, in Test 3, the difference between HCKD (13.89 ± 2.56 s) and AHKD (15.50 ± 3.08 s) was statistically significant and HCKD was measured more rapidly.

**Table 4:** Comparison between HCKD and AHKD

				unit: sec
	**HCKD** **M ± SD**	**AHKD** **M ± SD**	**t**	**p-value**
Test 1	12.36 ± 2.06	14.50 ± 2.80	-5.432	p < 0.001
Test 2	12.90 ± 2.70	14.61 ± 2.90	-3.671	0.001
Test 3	13.89 ± 2.56	15.50 ± 3.08	-4.127	p < 0.001

SD: standard deviation

**Figure 9.**
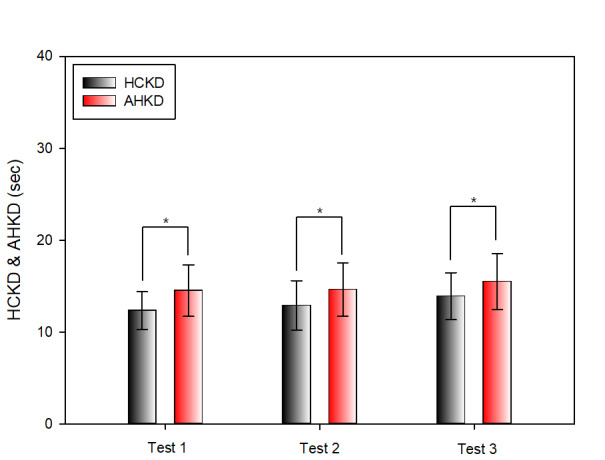
Comparison between HCKD and AHKD(*:p < 0.05)

#### Comparison of Results between VHKD and AHKD

Table 5 compares the results of Test 1, Test 2, and Test 3 of the K-D test between VHKD and AHKD. In Test 1, there was a significant difference between VHKD (22.24 ± 3.27 s) and AHKD (14.50 ± 2.80 s) and AHKD was measured more rapidly. In Test 2, there was a statistically significant difference between VHKD (23.23 ± 3.80 s) and AHKD (14.61 ± 2.90 s) and AHKD was measured more rapidly. In Test 3, the comparison between VHKD (24.04 ± 3.08 s) and AHKD (15.50 ± 3.08 s) yielded statistically significant differences and AHKD was measured more rapidly.

**Table 5:** Comparison between VHKD and AHKD

				unit: sec
	**VHKD** **M ± SD**	**AHKD** **M ± SD**	**t**	**p-value**
Test 1	22.24 ± 3.27	14.50 ± 2.80	13.649	p < 0.001
Test 2	23.23 ± 3.80	14.61 ± 2.90	12.878	p < 0.001
Test 3	24.04 ± 3.88	15.50 ± 3.08	15.055	p < 0.001

SD: standard deviation

**Figure 10.**
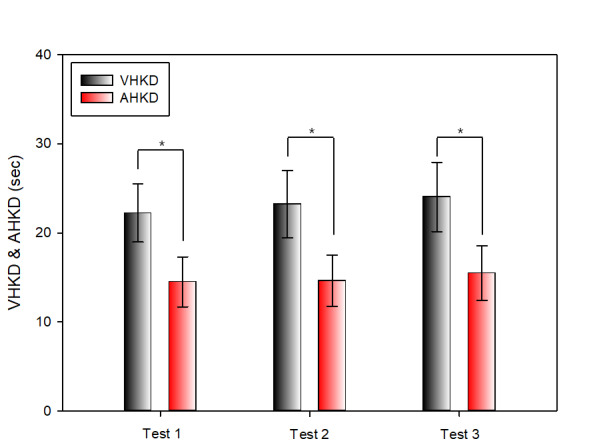
Comparison between VHKD and AHKD(*: p < 0.05)

### Correlation between K-D tests

#### Correlation between HCKD and VHKD

Tables 6, 7 and 8 show the correlation between HCKD and VHKD for Test 1, Test 2, and Test 3, respectively. In the correlation analysis between HCKD and VHKD in Test 1, there was a weak positive correlation (0.3 ≥ R ≥ 0.1), but no statistical significance was found. Similarly, there was a weak positive (+) correlation (0.3 ≥ R ≥ 0.1) between HCKD and VHKD in Test 2, but no statistical significance was found. However, between HCKD and VHKD in Test 3, there was a statistically significant strong positive correlation (0.7 ≥ R ≥ 0.3), with statistical significance.

**Table 6:** Correlation between HCKD and VHKD *(Test 1)*

**variable**	**HCKD** **Test 1**	**VHKD** **Test 1**	**p-value**
HCKD Test 1	1		p < 0.001
VHKD Test 1	0.194 (0.304)	1	p < 0.001
			(*: p < 0.05)

**Table 7:** Correlation between HCKD and VHKD *(Test 2)*

**variable**	**HCKD** **Test 2**	**VHKD** **Test 2**	**p-value**
HCKD Test 2	1		p < 0.001
VHKD Test 2	0.211 (0.263)	1	p < 0.001
			(*: p < 0.05)

**Table 8:** Correlation between HCKD and VHKD *(Test 3)*

**variable**	**HCKD** **Test 3**	**VHKD** **Test 3**	**p-value**
HCKD Test 3	1		p < 0.001
VHKD Test 3	0.432* (0.017)	1	p < 0.001
			(*: p < 0.05)

**Figure 11.**
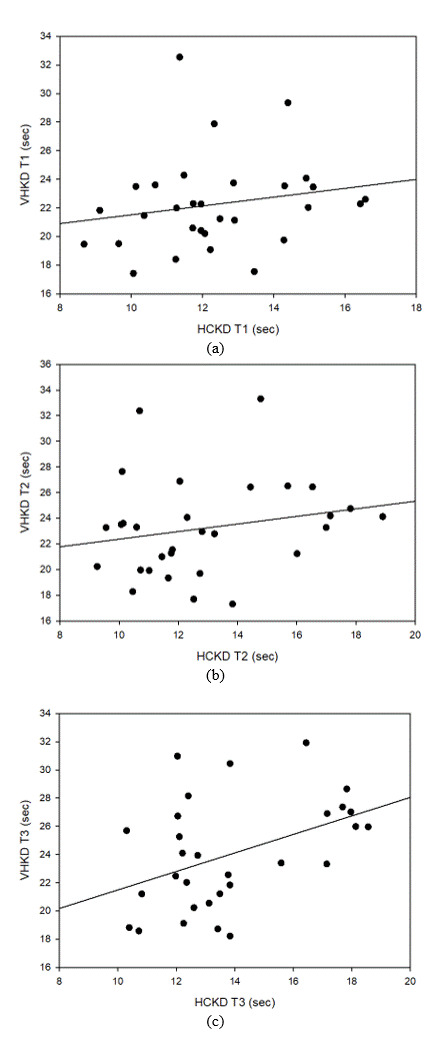
Correlation between HCKD and VHKD (a) Test 1, (b) Test 2, (c) Test 3

#### Correlation between HCKD and AHKD

Tables 9, 10, and 11 show the correlation between HCKD and AHKD for Test 1, Test 2, and Test 3, respectively. In the correlation analysis between HCKD and AHKD in Test 1, there was a strong positive correlation (0.7 ≥ r ≥ 0.3), which was statistically significant. In the correlation analysis between HCKD and AHKD in Test 2, there was also a strong positive correlation (0.7 ≥ r ≥ 0.3) that was statistically significant. Moreover, there was a very strong positive correlation between HCKD and AHKD in Test 3 (1.0 ≥ R ≥ 0.7), which was statistically significant.

**Table 9:** Correlation between HCKD and AHKD *(Test 1)*

**variable**	**HCKD** **Test 1**	**AHKD** **Test 1**	**p-value**
HCKD Test 1	1		p < 0.001
AHKD Test 1	0.641* (p < 0.001)	1	p < 0.001
			(*: p < 0.05)

**Table 10:** Correlation between HCKD and AHKD *(Test 2)*

**variable**	**HCKD** **Test 2**	**AHKD** **Test 2**	**p-value**
HCKD Test 2	1		p < 0.001
AHKD Test 2	0.589* (0.001)	1	p < 0.001
			(*: p < 0.05)

**Table 11:** Correlation between HCKD and AHKD *(Test 3)*

**variable**	**HCKD** **Test 3**	**AHKD** **Test 3**	**p-value**
HCKD Test 3	1		p < 0.001
AHKD Test 3	0.730* (p < 0.001)	1	p < 0.001
			(*: p<0.05)

**Figure 12.**
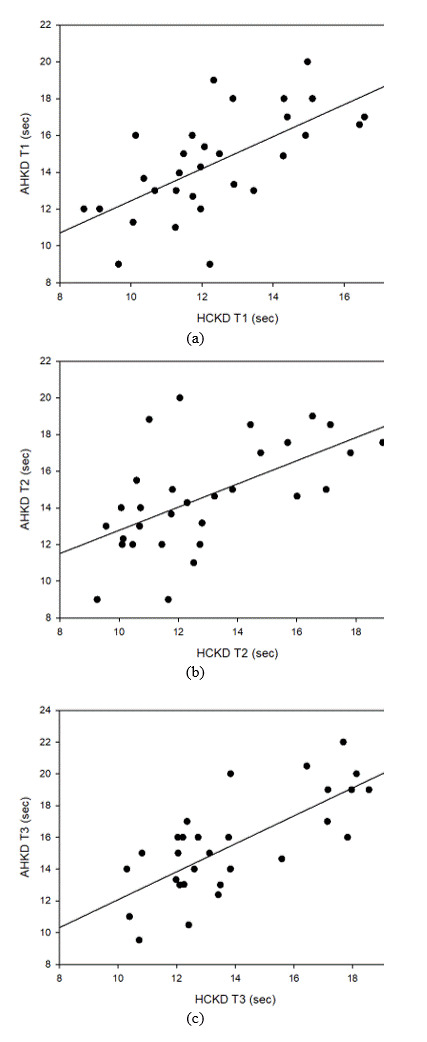
Correlation between HCKD and AHKD. (a) Test 1, (b) Test 2. (c) Test 3

#### Correlation between VHKD and AHKD

Tables 12, 13, and 14 show the correlation between VHKD and AHKD for Test 1, Test 2, and Test 3, respectively. In the correlation analysis between VHKD and AHKD for Test 1, there was a strong positive and statistically significant correlation (0.7 ≥ r ≥ 0.3). For Test 2, there was a strong positive correlation (0.7 ≥ r ≥ 0.3) between VHKD and AHKD, which was statistically significant. For Test 3, there was a very strong positive correlation (0.7 ≥ r ≥ 0.3) between VHKD and AHKD, which was statistically significant.

**Table 12:** Correlation between VHKD and AHKD *(Test 1)*

**variable**	**VHKD** **Test 1**	**AHKD** **Test 1**	**p-value**
VHKD Test 1	1		p < 0.001
AHKD Test 1	0.484* (0.007)	1	p < 0.001
			(*: p < 0.05)

**Table 13:** Correlation between VHKD and AHKD *(Test 2)*

**variable**	**VHKD** **Test 2**	**AHKD** **Test 2**	**p-value**
VHKD Test 2	1		p < 0.001
AHKD Test 2	0.427* (0.019)	1	p < 0.001
			(*: p < 0.05)

**Table 14:** Correlation between VHKD and AHKD *(Test 3)*

**variable**	**VHKD** **Test 3**	**AHKD** **Test 3**	**p-value**
VHKD Test 3	1		p < 0.001
AHKD Test 3	0.623* (p < 0.001)	1	p < 0.001
			(*: p < 0.05)

**Figure 13.**
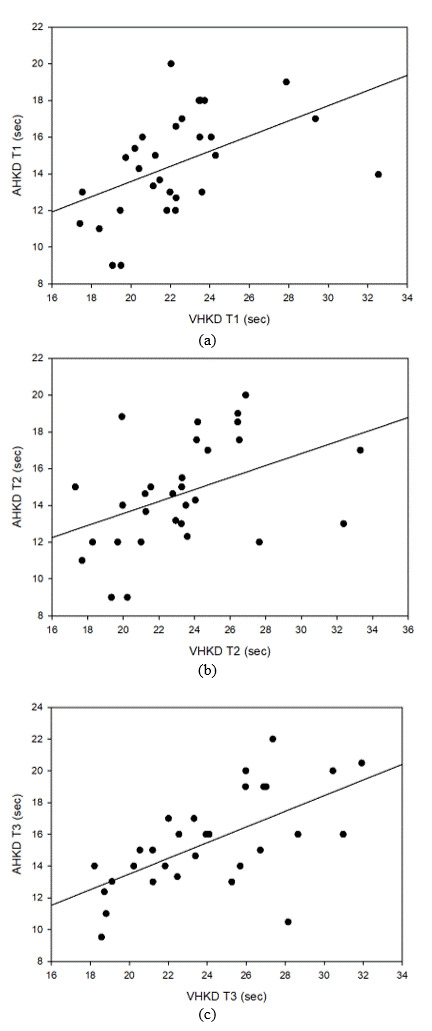
Correlation between VHKD and AHKD. (a) Test 1, (b) Test 2, (c) Test 3

### Error Frequency between K-D tests

#### Comparison of Error Frequency by Type in Test 1

Table 15 shows the results of the type-specific measurement errors (substitution [s], omission [o], addition [a], and transposition [t] errors) that occurred. There was no statistically significant difference in the frequency of errors of any type that occurred in Test 1 for each K-D chart.

**Table 15:** Frequency of error occurrence in Test 1

						unit: number
		**HCKD** **M ± SD**	**VHKD** **M ± SD**	**AHKD** **M ± SD**	**t**	**p-value**
s error	HCKD & VHKD HCKD & AHKD VHKD & AHKD	0.17 ± 0.46 0.17 ± 0.46 -	0.00 ± 0.00 - 0.00 ± 0.00	- 0.07 ± 0.37 0.07 ± 0.37	1.980 0.902 -1.000	0.057 0.375 0.326
o error	HCKD & VHKD HCKD & AHKD VHKD & AHKD	0.10 ± 0.40 0.10 ± 0.40 -	0.07 ± 0.37 - 0.07 ± 0.37	- 0.10 ± 0.31 0.10 ± 0.31	0.328 0.000 -0.372	0.745 1.000 0.712
a error	HCKD & VHKD HCKD & AHKD VHKD & AHKD	1.07 ± 1.39 1.07 ± 1.39 -	1.00 ± 1.34 - 1.00 ± 1.34	- 0.47 ± 0.90 0.47 ± 0.90	0.177 1.917 2.006	0.861 0.065 0.054
t error	HCKD & VHKD HCKD & AHKD VHKD & AHKD	0.03 ± 0.18 0.03 ± 0.18 -	0.00 ± 0.00 - 0.00 ± 0.00	- 0.07 ± 0.25 0.07 ± 0.25	1.000 -0.571 -1.439	0.326 0.573 0.161

SD: standard deviation (s): substitution error; (o): omission error; (a): addition error; (t): transposition error

**Figure 14.**
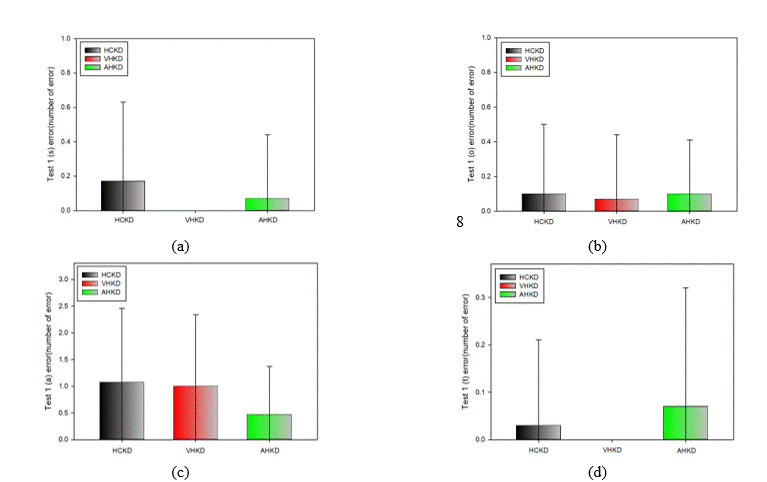
Frequency of error occurrence in Test 1(*: p < 0.05). (a) s error; (b) o error; (c) a error; (d) t error

#### Comparison of Error Frequency by Type in Test 2

Table 16 shows the results of the type-specific measurement errors in Test 2 for each K-D chart.

There was no statistically significant difference in the frequency of errors of any type in Test 2 for each K-D chart.

**Table 16:** Frequency of error occurrence in Test 2

						unit: number
		**HCKD** **M ± SD**	**VHKD** **M ± SD**	**AHKD** **M ± SD**	**t**	**p-value**
s error	HCKD & VHKD HCKD & AHKD VHKD & AHKD	0.07 ± 0.25 0.07 ± 0.25 -	0.10 ± 0.31 - 0.10 ± 0.31	- 0.10 ± 0.40 0.10 ± 0.40	-0.441 -0.372 0.000	0.662 0.712 1.000
o error	HCKD & VHKD HCKD & AHKD VHKD & AHKD	0.07 ± 0.37 0.07 ± 0.37 -	0.03 ± 0.18 - 0.03 ± 0.18	- 0.27 ± 1.11 0.27 ± 1.11	0.441 -0.972 -1.126	0.662 0.339 0.269
a error	HCKD & VHKD HCKD & AHKD VHKD & AHKD	0.83 ± 1.39 0.83 ± 1.39 -	0.60 ± 1.00 - 0.60 ± 1.00	- 0.37 ± 0.56 0.37 ± 0.56	0.690 1.919 1.045	0.495 0.065 0.305
t error	HCKD & VHKD HCKD & AHKD VHKD & AHKD	0.00 ± 0.00 0.00 ± 0.00 -	0.00 ± 0.00 - 0.00 ± 0.00	- 0.07 ± 0.25 0.07 ± 0.25	0.000 -1.439 -1.439	1.000 0.161 0.161

SD: standard deviation (s): substitution error; (o): omission error; (a): addition error; (t): transposition error

**Figure 15.**
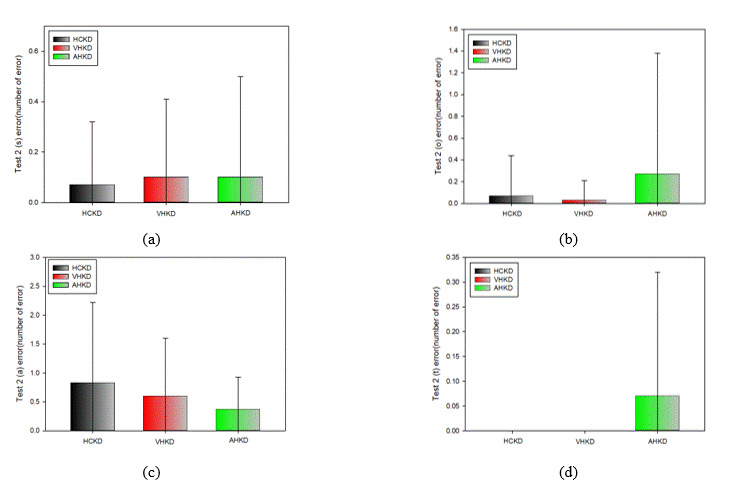
Frequency of error occurrence in Test 2(*: p < 0.05). (a) s error; (b) o error; (c) a error; (d) t error

#### Comparison of Error frequency by Type in Test 3

Table 17 shows the results of the type-specific measurement errors that occurred in Test 3 of each K-D chart.

There was no statistically significant difference in the frequency of errors of any type o that occurred in Test 3 of each K-D chart.

**Table 17:** Frequency of error occurrence in Test 3

						unit: number
		**HCKD** **M ± SD**	**VHKD** **M ± SD**	**AHKD** **M ± SD**	**t**	**p-value**
s error	HCKD & VHKD HCKD & AHKD VHKD & AHKD	0.03 ± 0.18 0.03 ± 0.18 -	0.10 ± 0.31 - 0.10 ± 0.31	- 0.07 ± 0.25 0.07 ± 0.25	-1.000 -0.571 0.571	0.326 0.573 0.573
o error	HCKD & VHKD HCKD & AHKD VHKD & AHKD	0.13 ± 0.51 0.13 ± 0.51 -	0.37 ± 1.40 - 0.37 ± 1.40	- 0.17 ± 0.91 0.17 ± 0.91	-0.839 -0.171 0.641	0.409 0.865 0.527
a error	HCKD & VHKD HCKD & AHKD VHKD & AHKD	0.53 ± 0.94 0.53 ± 0.94 -	0.70 ± 1.29 - 0.70 ± 1.29	- 0.43 ± 0.86 0.43 ± 0.86	-0.623 0.392 0.859	0.538 0.698 0.397
t error	HCKD & VHKD HCKD & AHKD VHKD & AHKD	0.03 ± 0.18 0.03 ± 0.18 -	0.03 ± 0.18 - 0.03 ± 0.18	- 0.03 ± 0.18 0.03 ± 0.18	0.000 0.000 0.000	1.000 1.000 1.000

SD: standard deviation (s): substitution error; (o): omission error; (a): addition error; (t): transposition error

**Figure 16.**
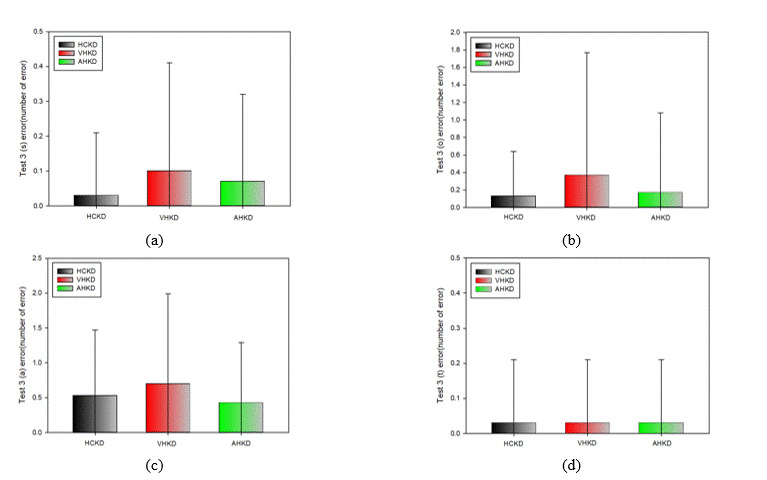
Frequency of error occurrence in Test 3(*: p < 0.05). (a) s error; (b) o error; (c) a error; (d) t error

**Table 18:** Results summary

**subjective symptoms**	**K-D test**	**K-D test** **correlation**	**K-D test** **error frequency**
①(Dizzy) statistical significance was appeared in the comparison of HCKD and VHKD and VHKD and AHKD. ② (Diplopia) no statistical significance was observed.	① In the case of Test 1,2,3, the comparison between HCKD and VHKD results was statistically significant and HCKD was measured fast..	① In the correlation analysis between HCKD and VHKD in Test 3, there was a strong positive (+) correlation (0.7≥r≥0.3), with statistical significance.	① There was no statistically significant difference in the frequency of errors for each type that occurred in Test 1 of each K-D chart.
③ (Definition) statistical significance was shown in the comparison of VHKD and AHKD. ④ (Legibility) statistical significance was shown in the comparison of VHKD and AHKD.	② In the case of Test 1,2,3, the comparison between HCKD and AHKD results was statistically significant and HCKD was measured fast.	② In the correlation analysis between HCKD and AHKD in Test 1,2,3, there was a strong positive (+) correlation (0.7≥r≥0.3), with statistical significance.	② There was no statistically significant difference in the frequency of errors for each type that occurred in Test 2 of each K-D chart.
⑤ (Discomfort) no statistical significance was observed. ⑥ (Ocular fatigue) statistical significance was shown in the comparison between HCKD and AHKD and between VHKD and AHKD.	③ In the case of Test 1,2,3, the comparison between VHKD and AHKD results was statistically significant and AHKD was measured fast..	③ In the correlation analysis between VHKD and AHKD in Test 1,2,3, there was a strong positive (+) correlation (0.7≥r≥0.3), with statistical significance.	③ There was no statistically significant difference in the frequency of errors for each type that occurred in Test 3 of each K-D chart.

## Discussion

Among human visual functions, dynamic vision that recognizes an external object in a moving state is a very important function. Among the types of dynamic visual acuity, saccadic eye movement is an important visual function from preschool age to adulthood, because it is closely related to reading ability. However, dynamic visual acuity tests are not standardized for static visual acuity.

Recently, VR/AR technology has increasingly been applied in various industries, such as defense, manufacturing, medicine, and education[24]. In this industry, VR/AR technology has converged on visual function test by a chart that is used in the medical field, and it is expected to be highly efficient compared to the current test system.

This study measured subjective symptoms in each test platform (hard copy, VR, AR), test record, test platform correlations, and error occurrence frequency. The dynamic visual acuity was measured and evaluated by the VR/AR dynamic visual acuity system.

### Subjective Symptom survey

In the subjective symptom survey results, there was a statistically significant difference in the comparison between HCKD and VHKD as well as between VHKD and AHKD in terms of dizziness, between VHKD and AHKD in terms of definition and legibility, and between HCKD and AHKD as well as between VHKD and AHKD in terms of ocular fatigue. No other comparisons yielded statistically significant differences. A level of appeal for subjective symptom for all items was found to be less than 1 point. Therefore, HCKD, VHKD, and AHKD did not enhance any subjective symptoms and the characteristics of each testing platform did not seem to have a significant effect on the test.

### Comparisons between K-D tests

Comparisons between K-D tests showed that Tests 1, 2, and 3 yielded statistically significant differences, and that HCKD was measured more rapidly than VHKD. For VHKD, since the testee has to look at the target by moving their body, he/she has to see the target with a reduced viewing angle through the lens, while the view of the external environment is blocked. HCKD was measured faster than VHKD because of the difference between looking at the target in a static state and looking at the target in a dynamic state. 

In the comparison between HCKD and AHKD, Test 1, 2, and 3 yielded statistically significant differences, and HCKD was again measured more rapidly. Compared with the comparison between HCKD and VHKD, the recording difference was significantly smaller. HCKD seemed to be measured faster than AHKD because the body is dynamic, and it is considered that AHKD measurement is closer to the HCKD record than VHKD, due to the nature of AHKD. 

In the comparison between VHKD and AHKD, Tests 1, 2, and 3 yielded statistically significantly different results, and AHKD was measured more rapidly. Although both VHKD and AHKD have the same goal of seeing the target by means of movement of the body; AHKD involves an environment in which the target is produced by a virtual graphic superimposed on a real environment. It may provide a faster record because AHKD is closer to the real environment than VHKD, in which the external environment is blocked. In the same context, AHKD is considered to be closer to HCKD than VHKD.

### Correlation analysis between K-D tests

In the correlation analysis between K-D tests, there was a positive correlation in all correlation analyses except for Tests 1 and 2 between HCKD and VHKD. This suggests that the test results of each participating platform can be obtained quickly on all test platforms. Due to the correlation, consistency between test platforms will be enhanced.

### Comparison of error frequency between K-D tests

Comparison of the error frequency between K-D tests showed no statistically significant difference in the error frequency between HCKD and VHKD, between HCKD and AHKD, or between VHKD and AHKD. There was no error specific to any test platform, and there was no significant difference in the types and frequency of errors among test platforms. The frequency of all errors was less than 1 on average, which means that the particular testing platform does not cause any error.

### Summary

In this paper, dynamic visual acuity measurement and its effectiveness were evaluated in VR/AR HMD environments through K-D tests performed in 3 environments. We verified the dynamic visual acuity measurement and its effectiveness in the VR/AR HMD environment by comparing subjective symptoms, K-D tests, correlations between K-D tests, and the frequency of errors between K-D tests for each environment. Compared with the HCKD test environment, the test in the VR and AR HMD environments, which are close to the real environment, seemed to be able to perform real environment-based measurement functions. However, in terms of the nature of the method used to implement the target, the AR method is more natural in conveying the human visual characteristics. With this method, confusion of the sense of depth is prevented, a real environment can be constructed without occlusion, and a real environment can be constructed without reduction of the viewing angle due to convex lens magnification, as compared to the VR method. In this regard, AHKD is closer to HCKD than VHKD in terms of experimental results. Implementation of a measurement system that is similar to real-life environments using the HMD is of great significance in terms of measuring accurate saccadic eye movements.

## Conclusions

The K-D test evaluates the accuracy, speed, and eye-tracking ability of saccadic eye movement. Other saccadic eye movement tests, including the standard K-D test, measure the visual acuity in a static state, in which the testees are seated and do not move their body, similar to a reading environment. In real life, visual activity often involves moving the body and external objects, but this current dynamic vision measurement systems do not incorporate this. The HMD-based K-D test chart presented in this study involves an environment in which participants should actually move their bodies, and in particular, AHKD can create a test environment close to the actual environment. This produces less confusion of the sense of depth, which can occur in VHKD because the vision is not blocked.

After applying the K-D test chart to VR HMD and AR HMD, the study verified the effectiveness and usability of VHKD and AHKD for implementing the K-D test method. This research can form the basis for a real environment-based dynamic visual acuity checking system using VR/AR element technology. In addition, it this research can be used as a basis for vision training by including immersive media technology.

## Acknowledgment

This research was supported by the Basic Science Research Program, through the National Research Foundation of Korea (NRF), funded by the Ministry of Education (2017R1A6A3A01007041) and the Ministry of Science, ICT, & Future Planning (MSIP) of the Korea Government (No. NRF-2017R1C1B5015194).
